# Evaluation of a research awareness training programme to support research involvement of older people with dementia and their care partners

**DOI:** 10.1111/hex.13096

**Published:** 2020-08-18

**Authors:** Jahanara Miah, Piers Dawes, Iracema Leroi, Bella Starling, Karina Lovell, Owen Price, Andrew Grundy, Suzanne Parsons

**Affiliations:** ^1^ Division of Neuroscience and Experimental Psychology University of Manchester Manchester UK; ^2^ Public Programmes Team, Research and Innovation Division Manchester University NHS Foundation Trust and the University of Manchester Manchester UK; ^3^ Manchester Centre for Audiology and Deafness (ManCAD) Manchester Academic Health Science Centre University of Manchester Manchester UK; ^4^ Department of Linguistics Macquarie University Sydney NSW Australia; ^5^ Global Brain Health Institute School of Medicine Trinity College Dublin Dublin 2 Ireland; ^6^ Division of Nursing Midwifery and Social Work Manchester UK; ^7^ School of Health Sciences University of Nottingham Nottingham UK

**Keywords:** dementia, older people, patient and public involvement, research awareness, training

## Abstract

**Background:**

Best‐practice guidelines recommend that appropriate support be provided to public contributors to facilitate their involvement in research. One form of support is research awareness training. Older people with dementia and care partners were involved in four Research User Groups (RUGs) in the UK, France, Cyprus and Greece. We delivered research awareness training (RAT) to the RUGs. The aim of this study was to evaluate the acceptability and perceived outcomes of the training from the perspective of RUG members.

**Methods:**

At the end of each research training session, participants completed the Training Acceptability Rating Scale‐section 2, which records the respondent's impressions of the training process and the outcomes of training. Participants were also invited to take part in semi‐structured interviews at the end of the programme.

**Results:**

Thirty‐four RUG members completed the TARS‐section 2 with 23 completing semi‐structured interviews. Over two‐thirds (67%) of participants rated their overall satisfaction with the RAT ‘a great deal’. Qualitative responses indicated that participants found group work to be beneficial for learning, the structure of training activities and topics covered appropriate. The type and format of the training materials were viewed as helpful, and they valued the new knowledge gained.

**Conclusions:**

The training contents were applicable, useful and relevant to the participants’ role within the research. We highlight the importance of facilitating participation by (a) fostering awareness of relevant research issues and (b) tailoring delivery of training according to the needs of the participants.

## 1 BACKGROUND

Patient and public involvement (PPI) in research is ‘doing research with or by the public, rather than to, about, or for them’.^1^ PPI recognizes the importance of patients and the public's viewpoints and that these views may differ from those of researchers.^1^, ^2^ PPI ensures research is appropriately designed with relevant outcomes and impact.^3^, ^4^ With this recognition, PPI is well established internationally through government policies, institutes and charities in the United States,^5^, ^6^ Canada,^7^ Europe,^8^, ^9^, ^10^, ^11^ the UK,^3^, ^12^ and Australia.^13^, ^14^

However, there is a debate on the need for PPI contributors to receiving research training.^15^, ^16^ Some consider that patients and the public are ‘experts by experience’ and so do not require training; training might professionalize or suppress the lay viewpoint and reduce the utility of the patients’ perspective by making it too similar to that of researchers.^17^, ^18^, ^19^ However, others assert that it is unreasonable to involve people in research without equipping them with the basic knowledge that facilities meaningful involvement.^20^ PPI is more likely to have a positive impact if PPI contributors receive appropriate training.^3^, ^16^, ^21^ If not, their contribution may be sub‐optimal and may contribute to, rather than reduce, research waste.^4^

Increasingly, people affected by dementia are involved in PPI role in research^22^, ^23^, ^24^, ^25^ and charities such as Alzheimer's society^24^, ^26^ are well established in PPI. Recent scoping reviews^22^, ^23^ and published evaluations and commentaries^23^, ^25^, ^27^, ^28^ highlight the impact of involvement in dementia. Furthermore, Alzheimer's Europe published a position paper^28^ on the PPI of people with dementia in research rationalizing the benefits and challenges in this area of work. PPI with people with dementia involves particular challenges around supporting memory and other cognitive and behavioural difficulties experienced by people living with dementia.^29^, ^30^, ^31^ There are particular challenges faced by people with dementia in PPI. These include a lack of training, not understanding the complexity and perceptions of research and confusion about the research process.^23^, ^25^ Therefore, basic level of research training should be available to PPI contributors,^32^ which may enable PPI contributors to share their viewpoints more efficiently^15^, ^16^ and shape the research in a meaningful way from posing the initial research questions to the final dissemination and implementation of the research outputs.^17^, ^33^ We aimed to understand the acceptability and perceived outcomes of the research awareness training from the perspective of patient and public advisors who received it as part of their PPI role in a multi‐national dementia research programme.

### 1.1 Study context

The current study is part of a work package dedicated to patient and public involvement embedded within the SENSE‐Cog,^34^ a 5‐year (2016‐2020) European multi‐site research programme investigating the combined impact of dementia, age‐related hearing and vision impairment. We set up four Research User Groups (RUGs) in Manchester (UK), Nice (France), Nicosia (Cyprus) and Athens (Greece) consisting of seven to ten older people with dementia and care partners in each site. RUGs were established to contribute PPI research activities in the running of the SENSE‐Cog programme. We delivered research awareness training (RAT) to RUG members to equip them with the skills and background knowledge required for involvement in the SENSE‐Cog programme. This paper describes the delivery and evaluation of RAT for older people with dementia and their care partners in role as PPI contributors.

### 1.2 Development of research awareness training

The RAT was developed as part of the Enhancing the Quality of User Involved Care Planning (EQUIP) programme to provide UK National Health Service mental health service users and care partners with an understanding of research and research terminology to support them as co‐researchers on a mental health research project.^35^, ^36^ EQUIP training was developed in partnership with service user and carers; therefore, an adaptation of this training was viewed appropriate for RUGs. The original EQUIP training involved a 6‐day course.^37^, ^38^ We consulted with RUGs^1^, ^16^ on their preferences on the delivery of the training (duration, frequency and practicalities). RUG members’ preference was for shorter, bite‐sized training delivered as needed every 3 months, as this facilitated participation of people with memory difficulties. We worked with the EQUIP team (KL, AG, OP) to modify the EQUIP training to 6 hour‐long sessions on key research topics that were relevant for RUGs to take part in the SENSE‐Cog PPI activities, see Bee at al^37^ for full details of the EQUIP training. We condensed parts of the information from chapter 1: research process, chapter 3: quantitative research design, chapter 4: quantitative data analysis, chapter 5: health economics, chapter 7: introduction to qualitative research methods and chapter 9: principles of ethical research. An example adaptation of chapter 7: health economics into RAT is illustrated in Appendix 1. Adaptation also included activities based on a theme that RUG members could related to, such as ‘planning a holiday’. We planned activities for pairs or small groups and ensured that the contents of each session could be delivered within 1 hour. The adapted training was structured to be delivered on an ‘as needed’ basis. For example, RAT on qualitative methods was offered immediately prior to the RUGs PPI activity reviewing a question related to qualitative aspects of SENSE‐Cog (Table 1).

**Table 1:** Research awareness training sessions

Session topic	Session content
Session 1: Research awareness	What is research Why research is important What are the different types of research (qualitative and quantitative) The importance of questioning research evidence
Session 2: Understanding the research process	Steps involved in the research process Group exercise How to read a paper and making sense of published papers Group exercise
Session 3: Qualitative Methods	What is qualitative research ‐ why? how? in what way? Examples of qualitative research Conducting interviews and qualitative data Advantages and disadvantages of using interviews Group exercise
Session 4: Quantitative Methods	What is quantitative research ‐ how much? how many? how often? to what extent? Steps in conducting randomized controlled trials (RCT) Examples of RCT Group exercise
Session 5: Developing and evaluating interventions	What is an intervention? Key elements of the development and evaluation of interventions Key questions in evaluating complex interventions Group exercise
Session 6: Health economics and Ethics & Governance	How do we make choices in health evaluation? Role of economic evaluation Group exercise Ethics and governance: approval requirements, how do we assess how ethical a research study is? Group exercise

### 1.3 We implemented the following approaches to support the delivery of the training

#### 1.3.1 Facilitation by experts

All RUGs were supported by a local PPI coordinator who was identified from among the research team in each site. Coordinators had a background in research with people with dementia (PwD) and experience of working with older adults in research settings. PPI coordinators were trained by the EQUIP team (KL, OP and AG who is a researcher with lived experience) at the University of Manchester based on a ‘train the trainers’ course^39^ to enable the PPI coordinators to deliver the RAT for local implementation. Each PPI coordinator was supported by up to two SENSE‐Cog staff members (researchers, research assistants) to help with the facilitation of the training and PPI activities, particularly with the PwD who did not have a care partner present.

#### 1.3.2 Individualized support

During introductory meetings, coordinators completed a support and learning needs form (Appendix 2) with individual RUG members to understand their needs to understand how to best facilitate their learning. Coordinators used this information to make individual support arrangements to facilitate each person's involvement. For example, for those with vision problems, coordinators positioned themselves close to the person and kept still while talking. People with vision problems were provided with training and RUG materials in large font black print on yellow paper. Requirements changed over time as people's needs changed. Coordinators checked people's support needs on an on‐going basis to ensure that appropriate support arrangements were in place.

#### 1.3.3 Interactive discussions

PPI coordinators delivered RAT to RUGs using interactive discussion. This involved an exchange of ideas where both facilitator and RUG members contributed to discussion of research topics. PPI coordinators used a variety of approaches for presenting key ideas, for example, interactive group work, role‐play exercises, case studies and pictures (Appendix 1).

#### 1.3.4 Informal discussion time

We scheduled informal meeting time before and after the training to encourage informal conversations. Informal meetings allowed RUG members to report, discuss any challenges or concerns and raise any issues after the main meeting. For example, RUG members may have required clarification of the involvement activity undertaken.

#### 1.3.5 Posting materials in advance of the training

We posted the meeting papers to RUG members 2 weeks before each session, to provide enough time to pre‐read the materials. Sending information ahead of the session allowed RUG members to make notes of their thoughts and identify anything that they did not understand before the training.

## 2 METHODS

### 2.1 Participants

The study participants were identified through the RUGs. Inclusion criteria were RUG membership, participation in RAT sessions and capacity to provide informed consent.^40^ We invited all RUG members (n = 34) who participated in RAT in Manchester, Nicosia, Nice and Athens to take part in the training evaluation. 34 RUG members consented to the TARS‐section 2 questionnaire evaluation and 23 consented to the semi‐structured interviews.

### 2.2 Design

We adopted a mixed methods approach. We used TARS‐section 2 and semi‐structured interviews to understand RUG members’ experience of the RAT. RAT sessions were delivered approximately every 3 months alongside RUG meetings over a 2‐year period. At the end of each session (Table 2), participants were asked to complete the TARS‐section 2 immediately after each session, to take account of those with memory problems.

**Table 2:** Time line of RAT delivery and TARS‐section 2 administration and semi‐structured interviews

Timeline	Jan 2017	April 2017	Jul 2017	Oct 2017	Jan 2018	April 2018	Oct/Nov 2018
Delivery of Research Awareness Training	✓	✓	✓	✓	✓	✓	
Administration of TARS‐section 2 questionnaires	✓	✓	✓	✓	✓	✓	
One to one interviews with RUG members							✓

### 2.3 Training acceptability rating scale (TARS)

The TARS is a self‐completed questionnaire which takes 5‐10 minutes to complete, consisting of two sections: TARS‐section 1 consists of six self‐report items, which measures training negative side effects, appropriateness, consistency and social validity. TARS‐section 2 focuses on the respondent's impressions of the training process and the outcomes of training and includes three open‐ended questions about ‘the most helpful’ parts of the training, ‘recommended changes’ and ‘any other comments’.^41^, ^42^ All numerical responses are rated on a four‐point Likert scale, ranging from ‘not at all' (score 1), ‘a little' (score 2), ‘quite a lot' (score 3) to ‘a great deal' (score 4). We did not use TARS‐section 1, in order to keep the survey simple to complete for RUG members and our key focus was on RUG members’ experience of training, rather than their view on social validity. We administered only TARS‐section 2 to focus on RUG members’ impressions of the training and the outcomes of training. Some wording of items was altered to make them applicable to the SENSE‐Cog RUG role (Appendix 3) and to make it understandable for the RUG members. For example, ‘Do you expect to make use of what you learnt in the training?’ was changed to ‘Do you think what you learnt in the training will be useful in your role as a RUG member?’. The TARS‐section 2 was translated into Greek and French using the ‘forward and back‐translation’ procedure for use in Nice, Nicosia and Athens.^43^

### 2.4 Semi‐structured interviews

RUG members who completed the RAT were invited take part in a semi‐structured interview (Appendix 4) to give their impressions of the training, indicate what knowledge and skills they had acquired and how they had applied the knowledge and skills. The semi‐structured interviews took place 6 months after the delivery of the last RAT session (Table 2). Coordinators conducted one‐to‐one semi‐structured interviews with RUG members in each site, and the interviews were audio recorded for transcription.^40^ Coordinators then translated the transcriptions into English for qualitative analysis.

### 2.5 Ethical considerations

The study received ethical approval from the Manchester University Research Ethics Committee. Additional ethical approvals were sought and obtained for each study site, relevant to local arrangements. Informed consent is an important consideration for research with people with dementia, particularly establishing whether potential participants have the capacity to provide informed consent and recognizing any changes in capacity that may develop as the research progresses.^44^, ^45^ The capacity of participants with cognitive impairment to give informed consent to participate was assessed on an on‐going basis by trained staff. Further details concerning on‐going assessment of capacity are available elsewhere.^40^

### 2.6 Data analysis

SPSS (IBM, Armonk NY) was used to generate descriptive statistics (frequencies, means, interquartile range and standard deviations) to describe the variables of interest. Interviews and free‐text data from the TARS‐section 2 open‐ended questions were analysed thematically.^46^ Data management was aided by the use of NVivo software version 11 (QSR International, Doncaster, Australia) and applying the Framework method.^47^ The Framework method allows in‐depth analysis of key themes across the whole data set, as well as between individual accounts using the interview topic guide (Appendix 4) as a starting point.^46^, ^47^ JM and SP independently examined the data to identify themes (Table S1: Codebook extract example). JM and SP then met and discussed the emerging themes to establish consensus for the interpretation of categories and themes. The emerging themes were then developed into a coding framework which included a list of themes with associated codes. The coding framework was emailed to coordinators in Nice, Nicosia and Athens to make suggestions for additional themes and/or combinations of themes. Any additional themes identified by coordinators were added to the list of themes. The overall data set was then analysed according to the final coding framework. We found the responses to the free‐text section of the TARS‐section 2 were very brief; mostly only few words. We therefore merged the TARS‐section 2 free‐text data and interview data to provide a richer understanding on emerging themes.

## 3 RESULTS

All 34 RUG members consented to participate in the evaluation using the TARS‐section 2^41^, ^42^ (Table 3: Characteristics of TARS‐section 2 participants across all sites). RUG members did not attend the training sessions consistently due to ill health, carer burden or hospital appointments and therefore RUG members did not complete the TARS‐section 2 for all sessions. Additionally, there were dropouts as some RUG members lost the capacity to provide informed consent (n = 2) or died (n = 5). 151 TARS‐section 2 questionnaires were completed over six training sessions across all sites. Participants included males (n = 9) and females (n = 14), aged 65‐85 years and included people with dementia (early‐stage dementia; n = 20).

**Table 3:** Characteristics of TARS‐section 2 participants across all sites

RUG Site	Person with dementia		Care partner		Total
	male	female	male	female	
Manchester	4	1	0	4	9
Nicosia	3	2	1	1	7
Nice	2	1	1	3	7
Athens	4	3	0	4	11
Total	13	7	2	12	34

### 3.1 Training acceptability rating scale

**Figure 1:**
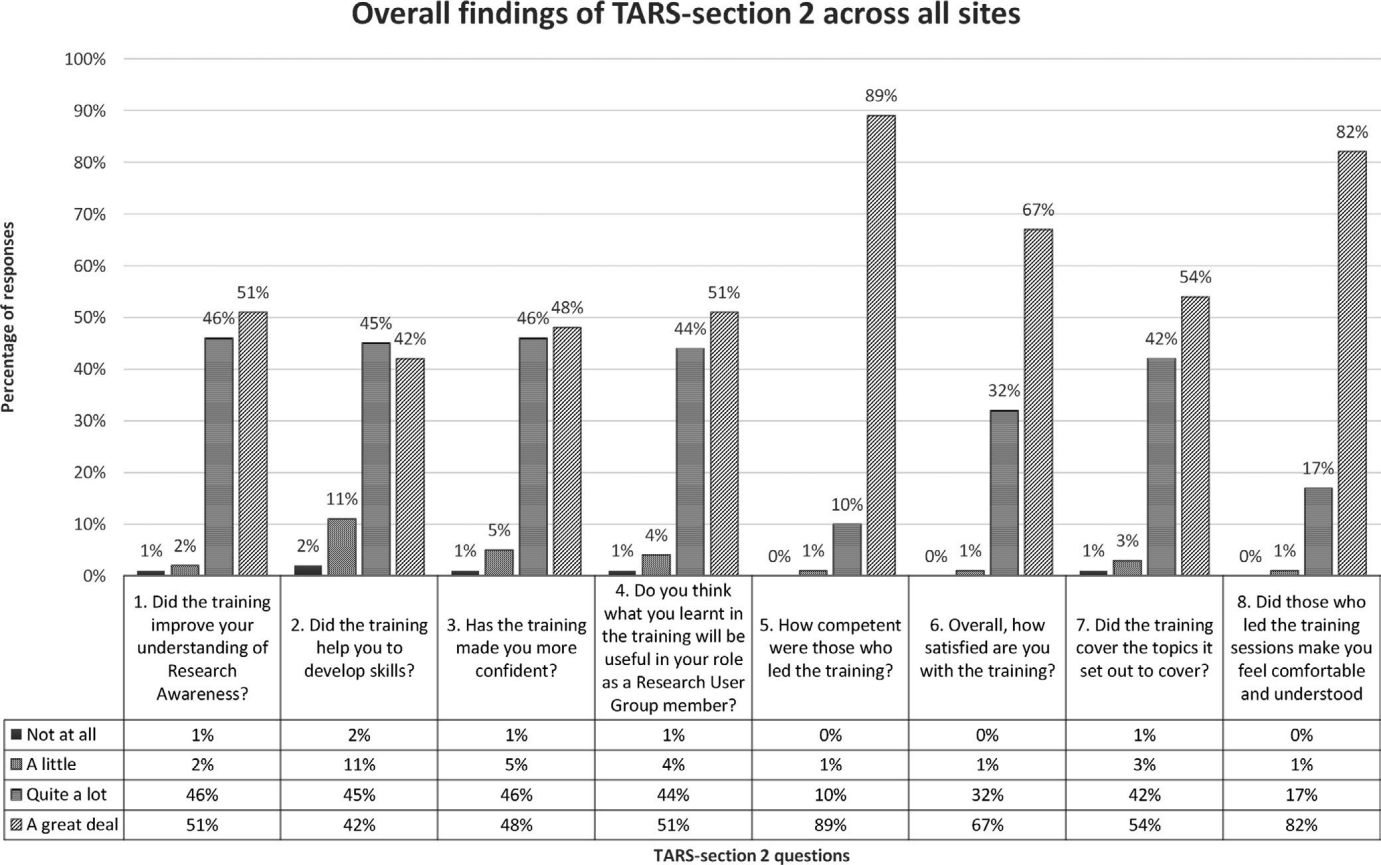
Overall findings of TARS‐section 2 across all sites

A majority of participants (51%, median score 3.5) rated the training at ‘a great deal’ in improving their understanding of research awareness (Figure 1). With 45% of participants, (median score of 3) viewing the training helped them ‘quite a lot’ to develop skills. Similarly, 48% of participants (median score of 3) answered ‘a great deal’ on the training increasing their confidence. A majority of participants (51%) rated ‘a great deal’ (median score of 4) regarding what they learnt in the training will be useful in their role as Research User Group members. The training facilitators were rated highly (89%, median score 4) at ‘a great deal’ as competent in leading the training. In terms of whether the training covered the topics it set out to cover, 54% (median score of 4) responded ‘a great deal’ and 82% of participants (median score of 4) rated ‘a great deal’ in facilitators making them feel comfortable and understood during the training sessions. All sites rated ‘a great deal’ (median score of 4) for overall satisfaction with the training and across all research awareness training sessions (median score 4). Further information on TARS‐section 2 scores descriptive statistics by sites is available in Table S2 and by RAT sessions available in Table S3.

In the Manchester site, one participant rated several questions at ‘not at all’, within the free‐text question they commented, ‘I’m a graduate in natural sciences, so I am already familiar with the content of the training’ (Manchester T130). Also, for question two (‘Did the training help you to develop skills?’), three participants rated ‘not at all’ in Manchester in relation to quantitative methods sessions in randomized controlled studies.

### 3.2 Qualitative results

Twenty‐three RUG members (Table 4: characteristics of interview participants across all sites) consented to the semi‐structured interviews. Reasons for non‐consenting for semi‐structure interviews were increased burden, ill health of either the PwD or the care partner. Interview participants included males (n = 9) and females (n = 14), aged 65‐85 years and included people with dementia (early‐stage dementia; n = 11).

**Table 4:** Characteristics of interview participants across all sites

RUG Site	Person with dementia		Care partner		Total
	male	female	male	female	
Manchester	3	0	0	4	7
Nicosia	2	1	1	1	5
Nice	1	1	0	3	5
Athens	2	1	0	3	6
Total	8	3	1	11	23

Not all respondents answered the open‐ended questions in the final section of the 151 TARS‐section 2 completed (Table S4). In the following quotes, the site is reported first (ie ‘Manchester’, ‘Nice’, ‘Nicosia’ or ‘Athens’). ‘T’ refers to TARS‐section 2 questionnaire or ‘I’ refers to semi‐structured interview response, TARS‐section 2 was completed anonymously; therefore, quotes do not specify whether it was a PwD or care partner. For semi‐structured interviews, quotes ‘RUG (number)’ refer to the individual RUG member being quoted. ‘PwD’ and ‘care partner’ refer to a person with dementia or a carer for someone with dementia, respectively. The counts of the themes from the TARS‐section 2 and semi‐structured interview are provided as supplementary information (Table S5).

Five themes developed from the analysis of the transcribed interviews with the RUG members: structuring of training activities alongside meetings, new knowledge, training materials and handouts, facilitator's role and approach, group work. It is important to distinguish that RUGs are not representative of the wider population, but instead their own distinctive cases.

### 3.3 Theme 1: Structuring of training activities alongside meetings

Participants talked about how the structuring of the training alongside the RUG meetings (for PPI activities) had been useful and had helped RUG members to contribute to the PPI activities:

‘It was interesting the fact that some group of people with no previous experience in such matters had an introduction of a research process and the detailed way it was delivered, the gradual way, seemed to me an awfully useful procedure. I think the training was a very good introduction in order someone to be able to participate as helpfully as possible in the research process’. (I, Athens, RUG 2, care partner)

‘The sessions (RAT training), the meetings went along so well that guided us through. It was not the case that one session was about something specific and then after six months the topic was something different in a way that we couldn’t participate…….extremely helpful and wonderful’. (I, Nicosia, RUG4, PwD)

‘For me the short trainings before starting to talk about the project are very good. It allows me to put my shoes in the subject’. (I, Nice, RUG 4, PwD)

A few participants mentioned how initially they were unsure why they required training in research, but over time it became apparent to them why it was structured in that manner:

‘I tried to understand what this was about (er). Especially, in the beginning when you focused on understanding ‘what is research’, I was wondering why we are working to understand ‘what is research’, if we are here for a study on hearing, memory and vision. (Er). At the end, the reason why was very clear. It became clear because we had talk about how do we evaluate a research study, how do we understand the study’. (I, Nicosia, RUG3, PwD)

### 3.4 Theme 2: New knowledge

Participants perceived that they had acquired new knowledge, participants talked about gaining an understanding of research procedures, understanding research papers, understanding how interventions are designed and trialled, and insight into ethics and governance of research:

‘I’ve learnt about things that I didn’t know about, like how they do the interventions and how you should question research studies, erm not just believe what they say, that we should look at what methods they used and who they recruited and how it was tested, all them things, I sound like I know it all now, don’t I’? (I, Manchester, RUG 4, PwD)

### 3.5 Theme 3: Training materials and handouts

RUG members indicated how the creative approaches of the training materials used supported their learning. Participants talked particularly about the holiday‐themed training activities. For example, RUG members were asked to plan a holiday using the research process, perform one‐to‐one interviews and use a paper survey to collect data on holiday experiences and compare different holiday packages with a set budget to understand the cost‐benefit analysis process:

‘So, and we did one thing on holidays, didn’t we? With James (PPI coordinator) and he…I can’t recall the reason why we were at one of the community centres and we were doing the holiday budget and…et cetera. And I can’t really recall the reason why he said to do it, but we act…we re‐enacted with each other, the people that were there. And it was…that was very interesting’ (I, Manchester, RUG3, PwD).

‘They made it fun as well as educational….I found that brilliant, that. I liked the activities with, you know, what they were doing and we were doing that thing on holidays and then putting them plans together and where this goes and why would you do that. When a few of us all sat round, I enjoyed that because I found it…you could all participate and put your own input into it. Somebody would say something you hadn't thought of. But I really enjoyed that activity one and the visual one; I thought that was really good. I did enjoy that…..When it was visual and you could see things, because I can't hear everything, I understood more because I could see it’. (I, Manchester, RUG2, ex‐ care partner)

Participants also talked about how information was presented to them using visual materials and large texts. Printed information provided to supplement the training was viewed as useful to refer to during and after the training sessions. Also handouts allowed participants to pre‐prepare for training, take notes and to refer back to the material or notes to refer to at a later date:

‘I liked the handouts, I always used to find them helpful to look through, so if I didn’t always understand what was being said, I could look at the handouts and read it’. (I, Manchester, RUG5, PwD)

‘It was easy to read, I liked the images used, and the writing was always big and clear. It was good to get the handouts before the training or the meeting, it gave you time to look through things and also to come prepared knowing what to expect at the meetings, that was good’ (I, Manchester, RUG 6, care partner).

### 3.6 Theme 4: Facilitator's role and approach

Participants talked about the important role of the facilitators and approach in supporting the process of learning. Participants commented about the facilitators being helpful by providing ‘personal attention to answering questions about everything and anything together with a proper way of dealing with the agenda’ (Manchester T127), ‘to see that there are people prepared to try to make the situation better for carers as well’ (Manchester T140). One participant mentioned how the facilitator encouraged everyone in the meetings to participate, ‘discussion is open and vivid and all participants are encouraged to participate’ (Athens T15).

Participants talked about how the approach used by facilitators to present topics to the RUGs was helpful. Participants felt that the clearness of the facilitator's presentation helpful:

‘The presentation regarding the topic was thorough and understandable, initially giving a good idea for the topic "research"’ (Athens, T2).

‘… the coordinators were always there to explain things that we didn’t understand, stopping at different times to make sure everyone are still with it but it wasn’t a patronizing thing, the conversations were always two way and I think we were always listened to’. (I, Manchester, RUG 7, care partner)

‘They made it fun to learn, … you didn’t make us feel like we didn’t know anything, we were encouraged to talk about it and make sense of it and ask questions about things that we didn’t understand’ (I, Manchester, RUG 6, care partner).

### 3.7 Theme 5: Group work

Group work was viewed as a positive experience for learning. Stimulating discussions in the group settings allow for the two‐way process of shared learning and personal development. Participants commented that group work made them feel part of the team and nurtured peer support for participants, which was viewed as a valuable way to help understand each other's circumstances and needs to support each other:

‘This was something innovative for me, to be participating in a group for research. In this sense, I will agree that even just participating in such a process is important…Yes, for sure. Otherwise, I wouldn’t be here today. It’s a good feeling, to be heard. You gain this feeling that you are not alone and that there are people that care for you, and that what you say can go further’ (Athens RUG 5, care partner).

‘I like coming to the meetings it does help me take my mind off things……So for me, I think yes, it was useful because it made my time worthwhile, giving my time to this group. But even if it wasn’t I enjoy coming to the groups, meeting other people and talking to them is good enough for me…..It’s also a good way for us to get together with other people in similar situation, to talk to others’ (I, Manchester, RUG 4, PwD).

### 3.8 TARS‐section 2 open‐ended responses on improvements and changes to training

Participants suggestions for improvements to training were varied, such as ‘a different approach between the care recipients and caregivers’ (Athens T19), ‘there is the need for more time on activities’ (Athens T50), ‘we would like more exercises’ (Athens T13). Others suggested shorter sessions to ‘start earlier and finish earlier’ (Manchester, T132) and one participant commented ‘sometimes too much information’ (Manchester, T147).

## 4 DISCUSSION

The purpose of this study was to understand the acceptability and perceived outcomes of RAT from the point of view of the RUG members who received it. Although we did not specifically evaluate which RAT elements were key in supporting RUG members’ involvement, participants rated acceptability highly and the perceived impact of the RAT as positive in general. Participants reported improvement in their knowledge of research. Participants viewed that the structuring of the training session delivery alongside the RUG meetings as relevant and supported their PPI role and found the opportunity to put their learning into practice with the PPI activities within the RUG meetings. Therefore, the provision of research knowledge may be particularly valuable in supporting PPI in research^18^, ^48^ and content of training should focus on providing knowledge that PPI contributors lack.^49^ One participant rated RAT as ‘not at all’ helpful, due to his previous knowledge on research through having completed a science degree. This highlights the need for further work on how training can be individualized or how experienced individuals can be involved in a group of people with mixed ability.

Participants perceived social interaction with learning as an important factor. This finding underlines the importance of the social aspects of RAT delivery. The delivery of training in group settings provided opportunities for RUG participants to share experiences and information with others. Our findings follow studies,^18^, ^48^, ^50^ which identified that the learning setting critically encourages discussions between learners and enhances the learning experience. This approach values RUG members bringing their direct, personal experience of the topic knowledge to the research process and a two‐way process of mutual learning. The qualitative findings showed that participants highly favoured group work, in particular the discussions.

Facilitation skills and competencies of the person delivering the training were important. In this study, the PPI coordinators had a background in research with people with dementia, which may have contributed to the positive rating of facilitators across sites. Being able to apply a range of didactic, small group and interactive approaches to training delivery was an essential skill for the person delivering the training. Facilitation of RAT requires the trainers to demonstrate knowledge and expertise in the training subject areas.

There has been little research on the recipients’ experience of PPI training. There is also debate on whether RAT is appropriate for PPI.^12^, ^16^, ^18^ Some have argued that providing training to PPI contributors undermines the validity of lay people's contribution to research.^17^, ^18^, ^33^ But lack of training support may be a barrier to effective PPI.

### 4.1 Strengths and limitations

The combination of quantitative and qualitative aspects in the TARS‐section 2 enabled us to evaluate the overall satisfaction and perceived impact of the training, as well as providing an opportunity for participants to suggest where changes to the training could be made. However, the TARS‐section 2 open‐ended section lacked detailed responses from participants, typically consisting of only a few words of response. Furthermore, as TARS‐section 2 was anonymously completed, it limited us in following up queries to investigate particular issues highlighted by participants and we did not know if the same participants took part in the semi‐structured interviews.

The TARS‐section 2 was completed immediately after each training session to ensure that those with memory problems were able to provide immediate feedback. However, administering the TARS‐section 2 only after RAT meant that we had no baseline information to understand the impact of the training pre‐ and post‐delivery and no data on the long‐term impact and acceptability of RAT were available. However, additional semi‐structured interviews with RUG members allowed further insight into the perceived impact of the RAT and factors that helped the learning experience.

We did not collect additional demographic data such as diagnosis, language proficiency, memory capabilities, educational level or other demographic details. Collecting additional demographic information could help with understanding differences and needs between individuals. In addition, we did not collect baseline data on RUG members’ research knowledge before the training, as there were no criteria for research knowledge to participate in PPI activities. In hindsight, it may have been useful to understand if RUG members had previous learning about research outside of the RAT. Details on baseline levels of knowledge may help tailor training support appropriately for each person.

Although the TARS‐section 2 was anonymous, responses may have been identifiable to the coordinators who were collecting data because of the small size of each RUG. Interview data were collected by the coordinators rather than an independent researcher. Lack of anonymity of responses and researcher bias may have impacted on responses provided by respondents and resulted in responses being overly positive.

### 4.2 Reflection

Although we consulted with the RUGs in the planning of RAT, that is, frequency and duration and delivery point of RAT, a more active involvement of RUG members in the development of the RAT content or exploring the options for different approaches for a particular activity could have improved the experiences of RUG members. We did not provide opportunities for RUG members to identify additional training needs that would be of interest to them. For future work, we will discuss research interests of PPI contributors in addition to the required training content considered appropriate by researchers for involvement of lay people in specific research questions.

The training session on ethics and governance was delivered in the last session because the other sessions were required to fit alongside specific SENSE‐Cog PPI activities. PPI Coordinators suggested that ethics and governance would have been more useful for RUG members to understand from the outset to provide the RUG members with an understanding of the standards and processes for ethical research.

## 5 CONCLUSION

RUG members reported that the training contents were applicable, useful and relevant to their involvement role in the research, fostered awareness of research and supported their involvement in research. This study demonstrates that the RAT package can be used to train older adults with dementia and their care partners. PPI contributors may be supported via facilitation by experts, individualized support, interactive discussions, informal discussion time and posting materials in advance of training.

Although PPI training takes time and resources, training plays a key part in supporting PPI contributors’ involvement in research.^6^, ^7^, ^10^, ^20^, ^21^, ^25^, ^26^, ^28^, ^37^ There is a need for funders to fund training and support for PPI contributors, and availability of tools for engaging patients and other stakeholders in research across conditions and populations.^6^, ^7^, ^10^, ^51^, ^52^

The RAT will be made available to researchers internationally to support PPI in research via application to Suzanne.Parsons@mft.nhs.uk. Additional research materials for the RAT can be obtained from the freely available online EQUIP training.^37^

## CONFLICT OF INTEREST

The authors declare that they have no competing interests.

## ETHICAL APPROVAL

The study was approved by the University of Manchester Research Ethics Committee (Reference Number 2017‐0627‐2142). Additional ethical approvals were sought and obtained for each study site (Nicosia, Nice, Athens), as relevant to local arrangements.

## Supporting information

Table S1

Table S2

Table S3

Table S4

Table S5

## Data Availability

The data sets generated and/or analysed during the current study are not publicly available due to the privacy of participants and risk of indirect identification by characteristics given in the interviews. TARS‐section 2 data are available by application to Suzanne.Parsons@mft.nhs.uk.

## APPENDIX 1

Slide 1

Slide 2

- Everyone uses economics every choice we make has benefits and costs.
- Every day we make lifestyle choices.
- Value of expected outcomes or benefits against some set of alternatives (alternative choice given up).
- Utility—preferences/satisfaction over some set of goods and services.

Slide 3

- Health‐care providers need to make choices and decisions.
- NHS—To choose between possible interventions, they calculate the Quality‐Adjusted Life Years (QALYs). Interventions costing the NHS less than £20 000 per QALY gained are cost‐effective. Those costing between £20 000 and £30 000 per QALY gained may also be deemed cost‐effective, if certain conditions are satisfied.
- Right prices—the cost of producing health‐care service/product.
- Decision on how to spend the health‐care budget—value for money.
- Cost‐effectiveness important factor in government decision making, but not the only factor. Fair distribution of health‐care resources (which is scarce).

Slide 4

- To choose between possible interventions.
- Decisions are driven by the perceived value of the outcome of the various options, what's the cost to society.
- Economics tries to find ways of understanding and quantifying that value and benefits and the utility (satisfaction).

Slide 5

- Time to be an economist!
- In small groups of 2‐3
- Complete exercise: Making choices
- Feedback to the whole group

Slide 6

Decision‐making tools help economist to consider benefits and costs of decision making.

Slide 7

Slide 8

- 3 star
- Travel from Gatwick
- Additional costs, time, stress of travelling from Manchester/might need overnight accommodation
- Risks: not making it on time—missing flight, queues/accident on motorway. Train delayed
- Advantage: 3 other people benefit from the holiday experience, you spend time with friends/ family, do activities together, etc

Slide 9

- 5 star
- Travel from Manchester
- Risks: You might fall ill or have an accident—you'll be on your own with no close friend or family to help you.
- Advantages: It 5 star signature holiday, transfer time only 20 minutes, nearby travel from Manchester, less travel time, fewer risks of missing flights. 2 weeks of just you time to relax and enjoy.
- Wrap up—making choices is difficult as there are different factors involved, one decision for one person might work well, but not for another.
- Decision on health‐care treatment and service—impacts on the individual (real cost of value).

## APPENDIX 2

### SUPPORT AND LEARNING NEEDS FORM

#### Research User Group (Site Name)

My Personal details:

**Table:**

Title and full name	
Contact details: what's the best way to contact you?	Postal Address	
	Contact Number	
	Email	
	Other	

My Needs and Support as a Research User Group member

**Table:**

My Needs and Support for Learning

**Table:**

Other, for example dietary requirements and travel arrangements

**Table:**

## APPENDIX 3

### ADAPTED TRAINING ACCEPTABILITY RATING SCALE (TARS)‐SECTION 2

#### Training Course: Adapted SENSE‐Cog Research Awareness Training

Date: Title of session:

Study Site:

These questions focus on how you feel today's session has gone. (ie whether you think the training was of a high quality, and whether you felt it was helpful or not).

For each question please circle the statement that best expresses your opinion.

PLEASE CIRCLE ONE ANSWER:

**Table:**

1	**Did the training improve your awareness of Research?**			
	Not at all	A little	Quite a lot	A great deal
2	**Did the training help you to develop skills to question Research?**			
	Not at all	A little	Quite a lot	A great deal
3	**Has the training made you more confident in talking about research?**			
	Not at all	A little	Quite a lot	A great deal
4	**Do you think what you learnt in the training will be useful in your role as a RUG member?**			
	Not at all	A little	Quite a lot	A great deal
5	**How competent were those who led the training?**			
	Not at all	A little	Quite a lot	A great deal
6	**Overall, how satisfied are you with the training?**			
	Not at all	A little	Quite a lot	A great deal
7	**Did the training cover the topics it set out to cover?**			
	Not at all	A little	Quite a lot	A great deal
8	**Did those who led the training sessions make you feel comfortable and understood**			
	Not at all	A little	Quite a lot	A great deal
9	**What was the most helpful part of the training for you, personally?**			
	
10	**What change, if any, would you recommend?** (eg to the content or teaching)			
	
11	**Please make any other comments that you would like to offer.**			
	**Thank you very much for your feedback**			

(from Milne, D. & Noone, S. 1996).

## APPENDIX 4

### RUG SEMI STRUCTURED INTERVIEW TOPIC GUIDE

*Questions to be paraphrased by patient and public involvement coordinators

1. What did you think about the Research Awareness Training?
2. Do you feel the Research Awareness Training sessions helped you in your role as a member of the Research User Group?
3. Did you feel that your thoughts/input were listened to and valued?
4. Did you feel that your thoughts/input where useful to the SENSE‐Cog research?
5. Were you given feedback from SENSE‐Cog researchers/coordinators on where the Research User Group member's had had an impact?
6. Do you feel your experience of being a Research User Group member matched up to how the role was originally described to you? Please explain:
7. To what extent do you feel you were able to contribute to the involvement tasks relating to the SENSE‐Cog programme?
8. To what extent do you feel your involvement:
  - Impacted on the different tasks within SENSE‐Cog?
  - Will impact on for the end users (older people with dementia and age‐related hearing and/or vision impairment)?
9. In terms of your role as a Research User Group member within SENSE‐Cog, to what extent do you feel you were:
  - Valued as a partner in this process?
  - Supported to get involved in the different tasks and opportunities within SENSE‐Cog?
10. Thinking about your involvement in the different tasks can you talk a bit about your relationship with:
  - The researchers, how they supported you and communicated with you?
  - The Research User Group coordinators, how they supported you and communicated with you?
