# Supplementary material for: Evaluation of a research awareness training programme to support research involvement of older people with dementia and their care partners
Source: Health Expect. 2020 Aug 18;23(5):1177–90. doi: 10.1111/hex.13096 (PMC7696121; doi:10.1111/hex.13096)
Supplement: Supplementary file 1 — Table S1 [file HEX-23-1177-s001.docx]

Supplementary Table S1: Codebook extract semi-structured interviews example

| Theme | Code | Definition | Example from manuscript |
| --- | --- | --- | --- |
| Structuring of training activities alongside meetings | Training alongside meetings | The delivery of training session’s straightaway preceding RUG meetings for PPI activities helped RUG members to contribute. | It was interesting the fact that some group of people with no previous experience in such matters had an introduction of a research process and the detailed way it was delivered, the gradual way, seemed to me an awfully useful procedure. I think the training was a very good introduction in order someone to be able to participate as helpfully as possible in the research process. (Athens RUG2)  To me personally, in the beginning it was interesting (as a topic) and then I became even more interested. First and foremost: the fact that we participate in a research process that takes place in parallel (with the training) and we are asked to understand/learn how it is exactly to do a research and how this research is useful for all individuals involved, and for all who have a role or a reason within the research to be interested at, was from the beginning a challenge. When it comes to my role in the team, at first I think I kept my distance to see how this is going to work, but I think that after that I had an active participation. I was trying to participate in my own way, to learn, to listen, to contribute and in general, when leaving the meetings I was thinking of them and I then communicated that with others, because as you know my husband was not (pause) (meaning: he wasn’t here in the meetings) (Athens RUG 5)  I thought the training and the research was very good. Because a lot of what was discussed and what told and what was taught, I hadn't realised, especially about the hearing and things like that, the sense that you don't' have. (Man RUG 2)  It made sense, especially having all that training before the main meeting, it crossed over nicely, and you can carry on with the conversation with the researchers, tell them what you think because you understand it better, does that make sense… you know what I mean, the training made you think about things and that was useful and then you can talk about it more easily with the researchers. I always found it interesting to listen to other people, sometimes they would say things that you didn’t think of and it’s good to understand things from a different point of view, I guess. (Man RUG6)  It was useful to know that information, it helped to understand things for the meetings that followed. I remember that hearing aid leaflet that we worked on, when them two ladies came to ask us to help them……to, to develop that leaflet. Yes, so the training we had before that helped me to prepare for that meeting, it was a natural follow on, and it made sense, and it was easier for me to talk about it. So yes, it has been helpful for this work. (Man RUG 7)  It (the training) helped us and it helped us in giving us ideas and allowed us to express our own ideas and experience for the meetings (RUG meetings) (Nicosia RUG1)  It became clear because we had talk about how do we evaluate a research study, how do we understand the study. We would like to thank you because we have gained a lot through this program (Nicosia RUG3)  “The sessions (RAT training), the meetings went along so well that guided us through. It was not the case that one session was about something specific and then after six months the topic was something different in a way that we couldn’t participate…….extremely helpful and wonderful.” (Nicosia, RUG4, PwD)  It was a very nice experience and very informative, because we have learnt a lot about us at a personal level and about how this research can reach other people who haven’t been part of the team. Especially for me who I am a carer of person with dementia, ..Yes, of course. The information helped to develop a clear idea about how this research is conducted and how is progressing…but when we talked about the research and as I we learned more, our interest for the next meeting was increased. However, as I said, with every meeting, the interest increased. …I think the information was quite helpful and it helped us a lot to think and gain knowledge on the research topic. (Nicosia RUG5)  yes, yes, very very useful….the trainings helps us to get more familiar with the topic, it makes more concrete for us what a research is, and clarifies our involvement. Because the word ‘research’ itself means all and nothing. (Nice RUG1)  For me the short trainings before starting to talk about the project are very good. It allows me to put my shoes in the subject. And actually it is true, there are topics more interesting than others. (Nice RUG 5) |
