# Supplementary material for: Evaluation of a research awareness training programme to support research involvement of older people with dementia and their care partners
Source: Health Expect. 2020 Aug 18;23(5):1177–90. doi: 10.1111/hex.13096 (PMC7696121; doi:10.1111/hex.13096)
Supplement: Supplementary file 2 — Table S2 [file HEX-23-1177-s002.docx]

Supplementary Table S2: TARS-section 2 scores descriptive statistics by sites

|  | | Did the training improve your understanding of Research Awareness? | Did the training help you to develop skills? | Has the training made you more confident? | Do you think what you learnt in the training will be useful in your role as a Research User Group member? | How competent were those who led the training? | Overall, how satisfied are you with the training? | Did the training cover the topics it set out to cover? | Did those who led the training sessions make you feel comfortable and understood |
| --- | --- | --- | --- | --- | --- | --- | --- | --- | --- |
| Manchester | N | 31 | 30 | 27 | 31 | 31 | 29 | 31 | 30 |
|  | Mean | 3.06 | 2.53 | 2.81 | 3.00 | 3.84 | 3.48 | 3.39 | 3.67 |
|  | Median | 3.00 | 3.00 | 3.00 | 3.00 | 4.00 | 4.00 | 3.00 | 4.00 |
|  | Std. Deviation | .680 | .776 | .681 | .683 | .454 | .574 | .667 | .479 |
| Nicosia | N | 35 | 34 | 35 | 35 | 35 | 35 | 35 | 35 |
|  | Mean | 3.51 | 3.50 | 3.63 | 3.71 | 3.94 | 3.69 | 3.60 | 3.91 |
|  | Median | 4.00 | 4.00 | 4.00 | 4.00 | 4.00 | 4.00 | 4.00 | 4.00 |
|  | Std. Deviation | .507 | .564 | .490 | .458 | .236 | .471 | .497 | .284 |
| Nice | N | 32 | 31 | 32 | 32 | 32 | 31 | 31 | 31 |
|  | Mean | 3.50 | 3.10 | 3.44 | 3.38 | 4.00 | 3.68 | 3.32 | 3.71 |
|  | Median | 4.00 | 3.00 | 3.50 | 3.00 | 4.00 | 4.00 | 3.00 | 4.00 |
|  | Std. Deviation | .568 | .651 | .619 | .609 | .000 | .475 | .702 | .529 |
| Athens | N | 53 | 53 | 53 | 52 | 53 | 53 | 53 | 53 |
|  | Mean | 3.66 | 3.62 | 3.57 | 3.60 | 3.79 | 3.75 | 3.60 | 3.91 |
|  | Median | 4.00 | 4.00 | 4.00 | 4.00 | 4.00 | 4.00 | 4.00 | 4.00 |
|  | Std. Deviation | .478 | .527 | .500 | .495 | .409 | .434 | .494 | .295 |
| Total | N | 151 | 148 | 147 | 150 | 151 | 148 | 150 | 149 |
|  | Mean | 3.47 | 3.26 | 3.41 | 3.45 | 3.88 | 3.67 | 3.50 | 3.82 |
|  | Median | 4.00 | 3.00 | 3.00 | 4.00 | 4.00 | 4.00 | 4.00 | 4.00 |
|  | Std. Deviation | .587 | .741 | .629 | .609 | .345 | .486 | .588 | .404 |
