# Supplementary material for: Evaluation of a research awareness training programme to support research involvement of older people with dementia and their care partners
Source: Health Expect. 2020 Aug 18;23(5):1177–90. doi: 10.1111/hex.13096 (PMC7696121; doi:10.1111/hex.13096)
Supplement: Supplementary file 3 — Table S3 [file HEX-23-1177-s003.docx]

Supplementary Table S3: TARS-section 2 scores descriptive statistics by RAT sessions

| Session | | Did the training improve your understanding of Research Awareness? | Did the training help you to develop skills? | Has the training made you more confident? | Do you think what you learnt in the training will be useful in your role as a Research User Group member? | How competent were those who led the training? | Overall, how satisfied are you with the training? | Did the training cover the topics it set out to cover? | Did those who led the training sessions make you feel comfortable and understood |
| --- | --- | --- | --- | --- | --- | --- | --- | --- | --- |
| Research awareness | N | 25 | 24 | 25 | 25 | 25 | 25 | 25 | 25 |
|  | Mean | 3.28 | 3.46 | 3.44 | 3.44 | 3.88 | 3.72 | 3.40 | 3.96 |
|  | Median | 3.00 | 3.50 | 3.00 | 3.00 | 4.00 | 4.00 | 3.00 | 4.00 |
|  | Std. Deviation | .542 | .588 | .507 | .583 | .332 | .458 | .577 | .200 |
| Understanding the process of research | N | 26 | 25 | 25 | 26 | 26 | 24 | 25 | 25 |
|  | Mean | 3.54 | 3.20 | 3.48 | 3.54 | 3.88 | 3.58 | 3.48 | 3.84 |
|  | Median | 4.00 | 3.00 | 3.00 | 4.00 | 4.00 | 4.00 | 3.00 | 4.00 |
|  | Std. Deviation | .508 | .816 | .510 | .508 | .326 | .504 | .510 | .374 |
| Qualitative Methods | N | 27 | 26 | 24 | 26 | 27 | 27 | 27 | 27 |
|  | Mean | 3.52 | 3.35 | 3.54 | 3.42 | 3.81 | 3.74 | 3.67 | 3.85 |
|  | Median | 4.00 | 3.00 | 4.00 | 4.00 | 4.00 | 4.00 | 4.00 | 4.00 |
|  | Std. Deviation | .700 | .745 | .658 | .758 | .483 | .447 | .480 | .362 |
| Quantitative Methods | N | 21 | 21 | 21 | 21 | 21 | 20 | 21 | 21 |
|  | Mean | 3.48 | 3.24 | 3.19 | 3.52 | 3.86 | 3.60 | 3.43 | 3.67 |
|  | Median | 3.00 | 3.00 | 3.00 | 4.00 | 4.00 | 4.00 | 3.00 | 4.00 |
|  | Std. Deviation | .512 | .625 | .814 | .512 | .359 | .503 | .598 | .483 |
| Developing interventions | N | 26 | 26 | 26 | 26 | 26 | 26 | 26 | 25 |
|  | Mean | 3.38 | 2.92 | 3.31 | 3.31 | 3.88 | 3.62 | 3.50 | 3.72 |
|  | Median | 3.50 | 3.00 | 3.00 | 3.00 | 4.00 | 4.00 | 4.00 | 4.00 |
|  | Std. Deviation | .697 | .977 | .736 | .736 | .326 | .571 | .707 | .458 |
| Health economics, Ethics & Governance | N | 26 | 26 | 26 | 26 | 26 | 26 | 26 | 26 |
|  | Mean | 3.62 | 3.42 | 3.50 | 3.50 | 3.96 | 3.73 | 3.50 | 3.85 |
|  | Median | 4.00 | 3.00 | 3.50 | 3.50 | 4.00 | 4.00 | 4.00 | 4.00 |
|  | Std. Deviation | .496 | .504 | .510 | .510 | .196 | .452 | .648 | .464 |
| Total | N | 151 | 148 | 147 | 150 | 151 | 148 | 150 | 149 |
|  | Mean | 3.47 | 3.26 | 3.41 | 3.45 | 3.88 | 3.67 | 3.50 | 3.82 |
|  | Median | 4.00 | 3.00 | 3.00 | 4.00 | 4.00 | 4.00 | 4.00 | 4.00 |
|  | Std. Deviation | .587 | .741 | .629 | .609 | .345 | .486 | .588 | .404 |
