# Supplementary material for: Evaluation of a research awareness training programme to support research involvement of older people with dementia and their care partners
Source: Health Expect. 2020 Aug 18;23(5):1177–90. doi: 10.1111/hex.13096 (PMC7696121; doi:10.1111/hex.13096)
Supplement: Supplementary file 4 — Table S4 [file HEX-23-1177-s004.docx]

Supplementary Table S4: Number of responses to the open-ended questions in TARS-section 2

| Open ended questions | Number of responses out of 151 for each question |
| --- | --- |
| Question 9 - What was the most helpful part of the training for you, personally? | 125 responses |
| Question 10 - What change, if any, would you recommend? (e.g. to the content or teaching) | 83 responses |
| Question 11 - Please make any other comments that you would like to offer. | 56 responses |
