# Supplementary material for: Evaluation of a research awareness training programme to support research involvement of older people with dementia and their care partners
Source: Health Expect. 2020 Aug 18;23(5):1177–90. doi: 10.1111/hex.13096 (PMC7696121; doi:10.1111/hex.13096)
Supplement: Supplementary file 5 — Table S5 [file HEX-23-1177-s005.docx]

Supplementary Table S5: Qualitative themes count

| Theme | Number of count in qualitative interviews | Number of count in TARS-section2 |
| --- | --- | --- |
| Structuring of training activities alongside meetings | 10 | 26 |
| New knowledge | 9 | 22 |
| Training materials and handouts | 8 | 18 |
| Facilitator’s role and approach | 11 | 27 |
| Group work | 9 | 32 |
